# The enhanced atorvastatin hepatotoxicity in diabetic rats was partly attributed to the upregulated hepatic Cyp3a and SLCO1B1

**DOI:** 10.1038/srep33072

**Published:** 2016-09-14

**Authors:** Nan Shu, Mengyue Hu, Zhaoli Ling, Peihua Liu, Fan Wang, Ping Xu, Zeyu Zhong, Binbin Sun, Mian Zhang, Feng Li, Qiushi Xie, Xiaodong Liu, Li Liu

**Affiliations:** 1 Center of Drug Metabolism and Pharmacokinetics, China Pharmaceutical University, Nanjing, China

## Abstract

Liver injury is a common adverse effect of atorvastatin. This study aimed to investigate atorvastatin-induced hepatotoxicity in diabetic rats induced by high-fat diet combined with streptozotocin. The results showed that 40 mg/kg atorvastatin was lethal to diabetic rats, whose mean survival time was 6.2 days. Severe liver injury also occurred in diabetic rats treated with 10 mg/kg and 20 mg/kg atorvastatin. The *in vitro* results indicated that atorvastatin cytotoxicity in hepatocytes of diabetic rats was more severe than normal and high-fat diet feeding rats. Expressions and activities of hepatic Cyp3a and SLCO1B1 were increased in diabetic rats, which were highly correlated with hepatotoxicity. Antioxidants (glutathione and N-Acetylcysteine), Cyp3a inhibitor ketoconazole and SLCO1B1 inhibitor gemfibrozil suppressed cytotoxicity and ROS formation in primary hepatocytes of diabetic rats. In HepG2 cells, up-regulations of CYP3A4 and SLCO1B1 potentiated hepatotoxicity and ROS generation, whereas knockdowns of CYP3A4 and SLCO1B1 as well as CYP3A4/SLCO1B1 inhibitions showed the opposite effects. Phenobarbital pretreatment was used to induce hepatic Cyp3a and SLCO1B1 in rats. Phenobarbital aggravated atorvastatin-induced hepatotoxicity, while decreased plasma exposure of atorvastatin. All these findings demonstrated that the upregulations of hepatic Cyp3a and SLCO1B1 in diabetic rats potentiated atorvastatin-induced hepatotoxicity via increasing ROS formation.

Diabetes mellitus is often accompanied by hypercholesterolemia which is thought to promote the development of atherosclerotic complications^1^. Statins are frequently prescribed to diabetic patients to reduce the cardiovascular risk^2^. However, clinical evidences have demonstrated that hepatotoxicity is one of the serious adverse effects of statins^3^,^4^,^5^. Previous studies have demonstrated that deficiency of coenzyme Q10 and selenoprotein modulation may be involved in statin-associated liver dysfunction^6^,^7^. Phosphorylation of Akt was reported to be a key indicator of susceptibility to statin-induced toxicity^8^.

Atorvastatin is metabolized to ortho- and para-hydroxy atorvastatin by Cyp3a/CYP3A4 in rat/human^9^,^10^, and it is also a substrate of SLCO1B1, whose important roles in hepatic elimination of atorvastatin have been identified^11^. Several reports demonstrated that CYP450 catalyzed substrate oxidation, accompanied by substantial formation of reactive oxygen species (ROS)^12^,^13^. Cyp3a2, the major Cyp450 enzyme isoform in rat liver, is reported to produce more ROS per amount than any other Cyp450 isoforms do^14^. Both clinical trials and animal experiments demonstrated that the expressions and activities of hepatic CYP450s^15^,^16^ and drug transporters^16^,^17^, may increase under diabetic condition leading to the alterations in hepatic drug dispositions. Thus we hypothesized that the upregulated hepatic Cyp3a and SLCO1B1 by diabetes may increase atorvastatin uptake and metabolism leading to massive ROS formation, which resulted in the enhanced atorvastatin-induced hepatotoxicity in diabetic rats.

The aims of the study were to investigate atorvastatin-induced liver injury in diabetic rats, and to illustrate the correlation between hepatotoxicity and the alterations of hepatic Cyp3a and SLCO1B1. Primary rat hepatocytes and HepG2 cells were used to verify the *in vivo* findings. Moreover, phenobarbital pre-treated rats were constructed to further demonstrate the coupling roles of Cyp3a and SLCO1B1 in atorvastatin-induced hepatotoxicity.

## Results

### Atorvastatin-induced hepatotoxicity in diabetic rats

The developed diabetic rats exhibited high levels of glucose, insulin, triglyceride and cholesterol in serum, accompanied by increased HOMA-IR. These syndromes were similar to those in type 2 diabetes patients, inferring that the developed diabetic rats may reflect the natural history and metabolic characteristics of human type 2 diabetes (Table 1). Atorvastatin treatment decreased serum levels of TC and TG, but following 5-day treatment, DM-AH rats began to die. On day 8, DM-AH rats all died accompanied by hepatomegaly and hepatorrhagia. The survival time of DM-AH rats was only 6.2 ± 0.9 days. Severe liver damage also occurred in DM-AL and DM-AM rats. As shown in Table 1, the liver weight (% of body weight) of DM-AM rats almost doubled than that of DM rats.

**Figure 1:**
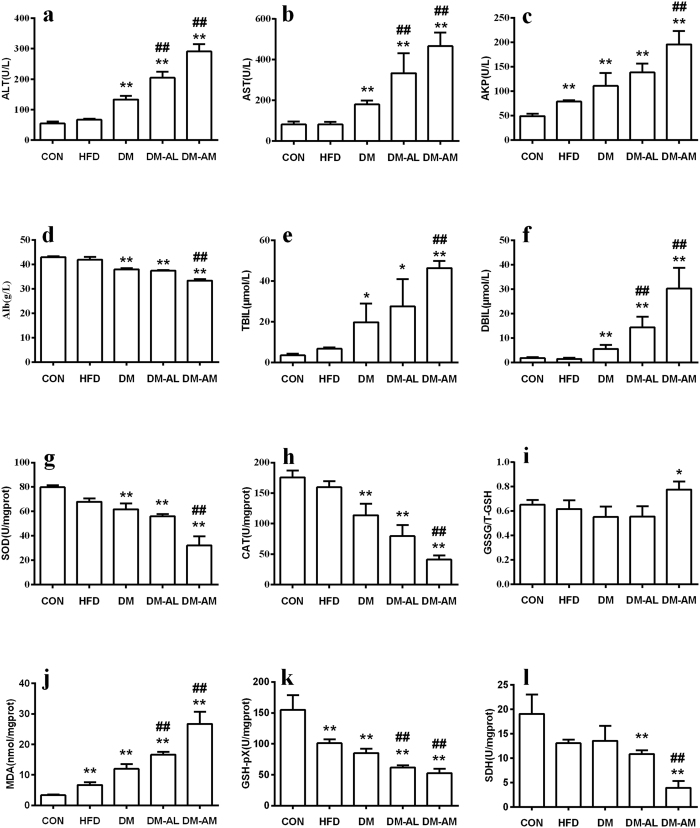
The levels of ALT (**a**), AST (**b**), AKP (**c**), Alb (**d**), TBIL (**e**), DBIL (**f**), SOD (**g**), CAT (**h**), GSSG/T-GSH (**i**), MDA (**j**), GSH-pX (**k**) and SDH (**l**) in normal rats (CON), rats fed with high-fat diet (HFD), diabetic rats (DM), DM rats treated with 10 mg/kg of atorvastatin (DM-AL) and 20 mg/kg of atorvastatin (DM-AM) for 10 days. Data are represented as means ± SD (n = 5) *p < 0.05, **p < 0.01 vs. CON, ^#^p < 0.05, ^##^p < 0.01 vs. DM.

**Figure 2:**
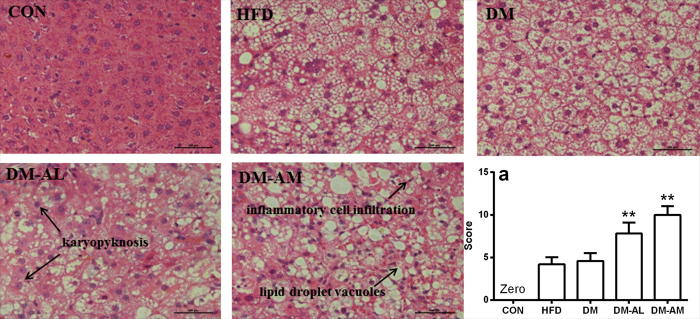
Histological analyses of livers from normal rats (CON), rats fed with high-fat diet (HFD), diabetic rats (DM), diabetic rats treated with 10 mg/kg atorvastatin (DM-AL) and 20 mg/kg atorvastatin (DM-AM). All sections were stained with hematoxylin-eosin (magnification, 200×). Scale bar represented 500 μm. Histopathological damage scores for all experiment rats (a). Data were represented as means ± SD (n = 5). *p < 0.05, **p < 0.01 vs. DM.

There were slightly increases in the levels of serum hepatotoxic biomarkers in DM rats, and atorvastatin treatment further elevated the levels of these biomarkers in a dose-dependent manner (Fig. 1a–f). Compared with DM rats, ALT, AST and AKP levels approximately doubled, TBIL and DBIL levels almost tripled in DM-AM rats. A significant decrease in level of Alb was observed in DM-AM rats. Histopathological examination showed that atorvastatin treatment aggravated fatty degeneration and vacuolization. DM-AL rats and DM-AM rats also showed karyopyknosis, necrosis, inflammatory cell infiltration and diffused hemorrhage (Fig. 2). Higher histopathological damage scores were observed in DM-AL rats and DM-AM rats, which was consistent with alterations in hepatotoxic biomarkers. All these results verified that atorvastatin could induce severe hepatic injury in diabetic rats.

### Hepatotoxicity of atorvastatin in primary rat hepatocytes

**Figure 3:**
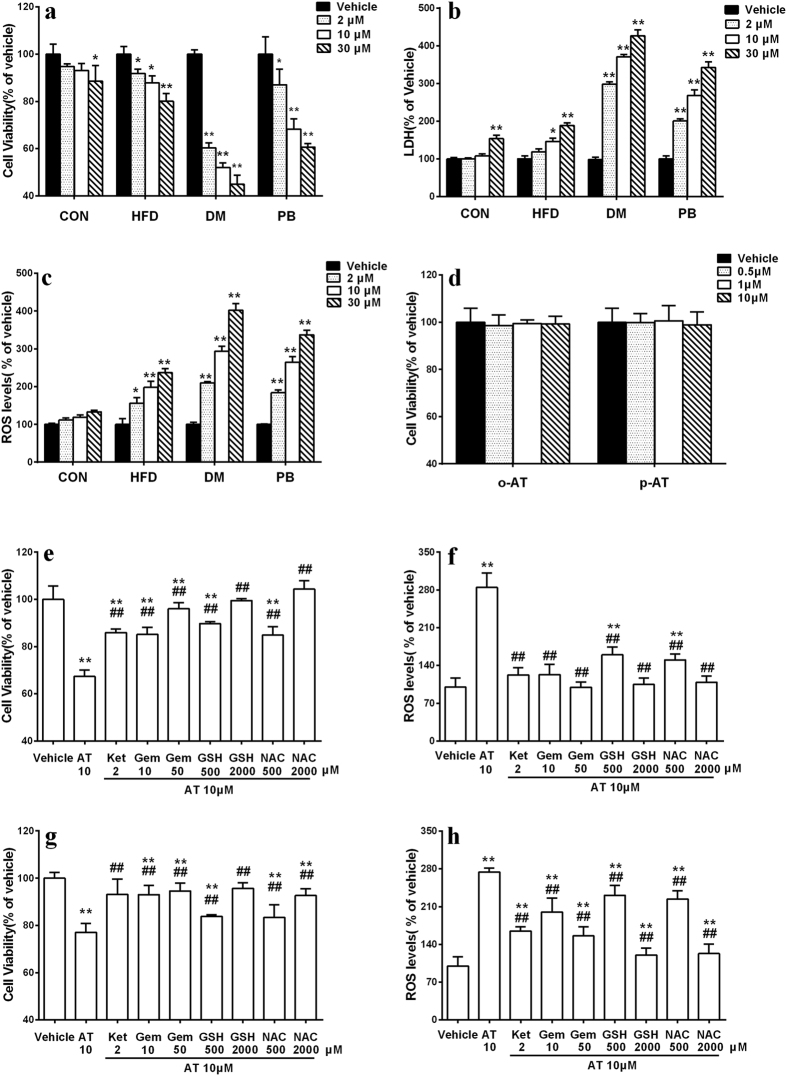
Cell viability (**a**) and LaDH release (**b**) following 96-h incubation with atorvastatin (AT) in primary hepatocytes of normal rats (CON), high-fat diet fed rats (HFD), diabetic rats (DM), phenobarbital pre-treated rats (PB). Effects of atorvastatin on the formation of ROS in primary rat hepatocytes (**c**); Effects of ketoconazole (Ket, 2 μM), gemfibrozil (Gem, 10 and 50 μM), glutathione (GSH, 0.5 and 2 mM) and N-Acetylcysteine (NAC, 0.5 and 2 mM) on the ROS formation and cytotoxicity induced by atorvastatin (10 μM) in primary hepatocytes of DM rat (**e,f**) and PB rats (**g,h**); The hepatotoxicity of ortho-hydroxy atorvastatin (o-AT) and para-hydroxy atorvastatin (p-AT) in primary hepatocytes of CON rats (**d**). Data were represented as means ± SD (n = 4). *p < 0.05, **p < 0.01 vs. vehicle, ^#^p < 0.05, ^##^p < 0.01 vs. AT.

As shown in Fig. 3a,b, atorvastatin-induced hepatotoxicity was dependent on pathophysiologic status of rats. Atorvastatin concentration-dependently induced hepatotoxicity in primary hepatocytes of DM rats, the cell viability after 30 μM treatment had a 50% decrease compared with vehicle treatment. Atorvastatin also induced hepatotoxicity in primary hepatocytes of CON and HFD rats, but the extent was slighter. These results demonstrated that atorvastatin-induced hepatotoxicity aggravated under diabetic status.

### Atorvastatin uptake and metabolism in primary rat hepatocytes

**Figure 4:**
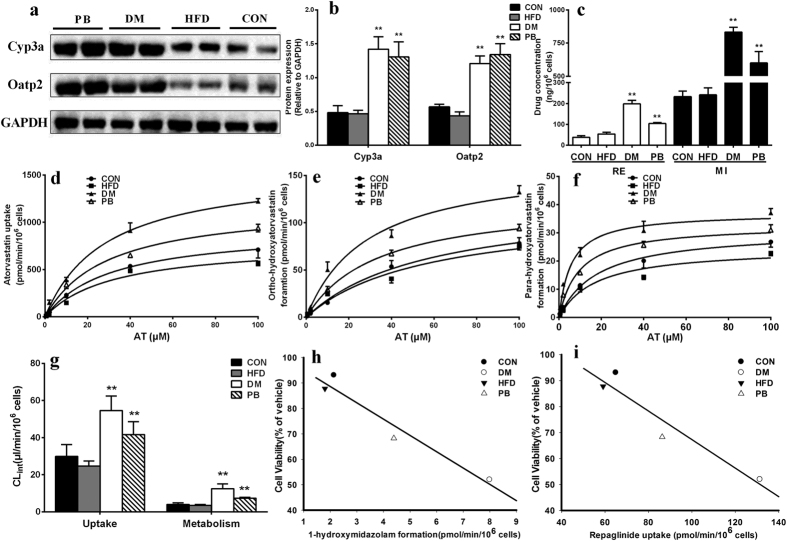
Relative protein levels (**a,b**) of Cyp3a and SLCO1B1 in normal rats (CON), rats fed with high-fat diet (HFD), diabetic rats (DM), normal rats treated with phenobarbital (PB); Repaglinide (20 μM) uptake and formation of 1-hydroxymidazolam from midazolam (10 μM) in hepatocytes of CON, HFD, DM and PB rats (**c**); Atorvastatin (0.5, 2, 10, 40, 100 μM) uptake (**d**) and formation of ortho-hydroxylation (**e**) and para-hydroxylation (**f**) in primary hepatocytes of CON, HFD, DM and PB rats; Uptake and metabolism intrinsic clearance of atorvastatin in primary hepatocytes of CON, HFD, DM and PB rats (**g**). Correlations between (**h**) Cyp3a activity (1-hydroxymidazolam formation), (**i**) SLCO1B1 activity (repaglinide uptake) and cell viability in primary hepatocytes of normal rats (CON), high-fat diet fed rats (HFD), diabetic rats (DM) and phenobarbital pre-treated rats (PB). Full-length blots are presented in the Supplement. Data are represented as mean ± SD (n = 4) *p < 0.05, **p < 0.01 vs. CON. Symbol: AT, atorvastatin, RE: repaglinide, MI: midazolam.

As illustrated in Fig. 4d, the uptake of atorvastatin by hepatocytes of DM rats was significantly higher than those by CON and HFD rats. Intrinsic clearances (CL_int_) of uptake in hepatocytes of CON, HFD and DM rats were estimated to be 29.89 ± 5.53, 24.61 ± 2.35 and 54.62 ± 6.73 μl/min/10^6^ cells, respectively (Fig. 4g). Atorvastatin metabolism was also evaluated (Fig. 4e,f). As expected, formations of the two metabolites in hepatocytes of DM rats were markedly higher than those of CON rats and HFD rats. The estimated total intrinsic clearances of the two metabolites in CON rats, HFD rats and DM rats were 3.95 ± 0.76, 3.51 ± 0.39 and 12.47 ± 2.20 μl/min/10^6^ cells, respectively (Fig. 4g). Activities of hepatic Cyp3a and SLCO1B1 were verified using the metabolism of midazolam and uptake of repalinide, respectively (Fig. 4c). Expressions of corresponding proteins were also measured (Fig. 4a,b). The results demonstrated that both expressions and activities of hepatic Cyp3a and SLCO1B1 siginificantly upregulated in diabetic rats.

Significant correlations between hepatotoxicity and Cyp3a activity (r = 0.955, p = 0.047) as well as SLCO1B1 activity (r = 0.970, p = 0.029) were observed (Fig. 4h,i), suggesting that the upregulated Cyp3a and SLCO1B1 may be responsible for the aggravated atorvastatin-induced hepatotoxicity under diabetic status.

To study whether the cytotoxic effects of atorvastatin resulted from its metabolites, cytotoxic effects of the two metabolites on primary hepatocytes of CON rats were investigated. As shown in Fig. 3d, the two metabolites showed no cytotoxicity high up to 10 μM. This excluded the contributions of the two metabolites to the atorvastatin-induced hepatotoxicity.

### Effect of atorvastatin on oxidative stress-related biomarkers

Oxidative stress-related parameters were measured (Fig. 1g–l). It was observed that atorvastatin dose-dependently lowered SOD, CAT, GSH-pX and SDH activities and increased MDA levels in liver. A significant increase in the level of GSSG/T-GSH was also observed in the liver of DM-AM rats when compared with DM rats. These results indicated that oxidative stress may be involved in the atorvastatin-induced hepatotoxicity in diabetic rats.

To further investigate the role of oxidative stress in hepatotoxicity, ROS level in primary hepatocytes was measured using DCFH-DA as a fluorescent probe. As depicted in Fig. 3c, atorvastatin concentration-dependently increased the generation of ROS. The strongest production of ROS occurred in hepatocytes of diabetic rats, which was in parallel to hepatotoxicity. These results suggested that excess production of ROS resulted in the hepatotoxicity. Antioxidants, GSH (0.5 and 2 mM) and N-Acetylcysteine (0.5 and 2 mM), SLCO1B1 inhibitor, gemfibrozil (10 and 50 μM) concentration-dependently reversed the atorvastatin-induced cytotoxicity and the production of ROS in primary hepatocytes of DM rats. Cyp3a inhibitor, ketoconazole (2 μM) showed similar effects (Fig. 3e,f). Higher concentration of ketoconazole (10 μM) was not used because of the cytotoxicity. These observations revealed that Cyp3a and SLCO1B1 were critical in the production of ROS.

### Contributions of CYP3A4 and SLCO1B1 to atorvastatin hepatotoxicity in HepG2 cells

**Figure 5:**
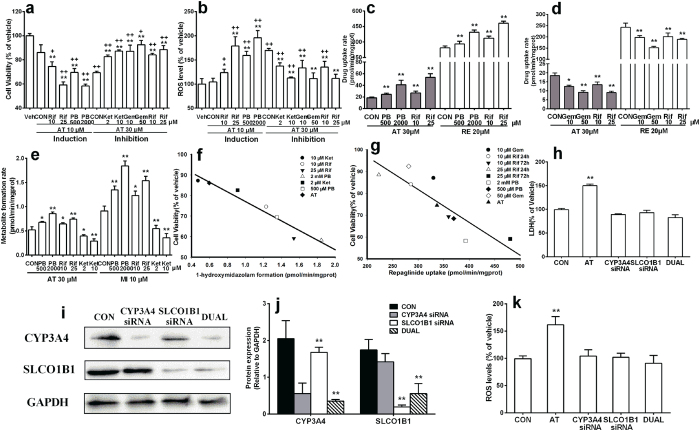
Effects of 72-h pre-incubation with CYP3A4/SLCO1B1 inducers (phenobarbital, PB and rifampicin, Rif) or 24-h co-incubation with CYP3A4 inhibitor (ketoconazole, Ket), SLCO1B1 inhibitors (gemfibrozil, Gem and rifampicin, Rif) on atorvastatin hepatotoxicity (**a**) and ROS generation (**b**) in HepG2 cells. Effects of SLCO1B1 inducers (**c**) and inhibitors (**d**) on atorvastatin and repaglinide (RE) uptake by HepG2 cells. Influences of CYP3A4 inducers and inhibitor on atorvastatin (AT) and midazolam (MI) metabolism (**e**). Correlations between (**f**) CYP3A4 activity (1-hydroxymidazolam formation), (**g**) SLCO1B1 activity (repaglinide uptake) and cell viability in HepG2 cells. Expressions of CYP3A4 and SLCO1B1 in HepG2 cells with CYP3A4, SLCO1B1 or dual knockdown (**i,j**). Effects of CYP3A4, SLCO1B1 or dual knockdown on atorvastatin hepatotoxicity (**h**) and ROS formation (**k**) in HepG2 cells. Full-length blots are presented in the Supplement. Data were represented as means ± SD (n = 4) *p < 0.05, **p < 0.01 vs. control cell, ^+^p < 0.05, ^++^p < 0.01 vs. vehicle.

The contributions of hepatic CYP3A4 and SLCO1B1 to atorvastatin hepatotoxicity were further investigated in HepG2 cells. Results showed that although 10 μM atorvastatin little affected the cell viability, 72-h pre-incubation with CYP3A4/SLCO1B1 inducers phenobarbital (0.5 and 2 mM) or rifampicin (10 and 25 μM) significantly increased atorvastatin-induced hepatotoxicity and ROS generation. On contrast, co-incubation with SLCO1B1 inhibitors (gemfibrozil and rifampicin) and CYP3A4 inhibitor ketoconazole concentration-dependently alleviated the atorvastatin-induced hepatotoxicity and ROS generation (Fig. 5a,b).

Atorvastatin uptake and metabolism in HepG2 cells were assessed. Midazolam and repaglinide were used to index the activities of CYP3A4 and SLCO1B1 (Fig. 5c–e), respectively. It was consistent with our expectation that 72-h pre-treatment with phenobarbital or rifampicin significantly increased atorvastatin uptake and metabolism in HepG2 cells in a concentration-dependent manner, while co-incubation with ketoconazole concentration-dependently inhibited atorvastatin metabolism in HepG2 cells. Similarly, co-incubation with SLCO1B1 inhibitors gemfibrozil or rifampicin concentration-dependently inhibited atorvastatin uptake by HepG2 cells. Significant correlations between atorvastatin hepatotoxicity and activities of CYP3A4 (r = 0.968, p = 0.0003) and SLCO1B1 (r = 0.781, p = 0.0131) in HepG2 cells were also found (Fig. 5f,g).

Effects of siRNA-mediated knockdown of CYP3A4 and SLCO1B1 on atorvastatin hepatotoxicity in HepG2 cells were further performed to verify contributions of CYP3A4 and SLCO1B1 to atorvastatin hepatotoxicity. Efficiencies of knockdowns were confirmed by analyses of CYP3A4 and SLCO1B1 protein levels (Fig. 5i,j). It was consistent with our expectation that CYP3A4 and SLCO1B1 knockdown attenuated atorvastatin hepatotoxicity in HepG2 cells, as well as the increased ROS level (Fig. 5h,k). Together with previous induction and inhibition studies, all the results demonstrated the pivotal roles of CYP3A4 and SLCO1B1 in atorvastatin hepatotoxicity.

### Atorvastatin-induced hepatotoxicity in rats pre-treated with phenobarbital

**Figure 6:**
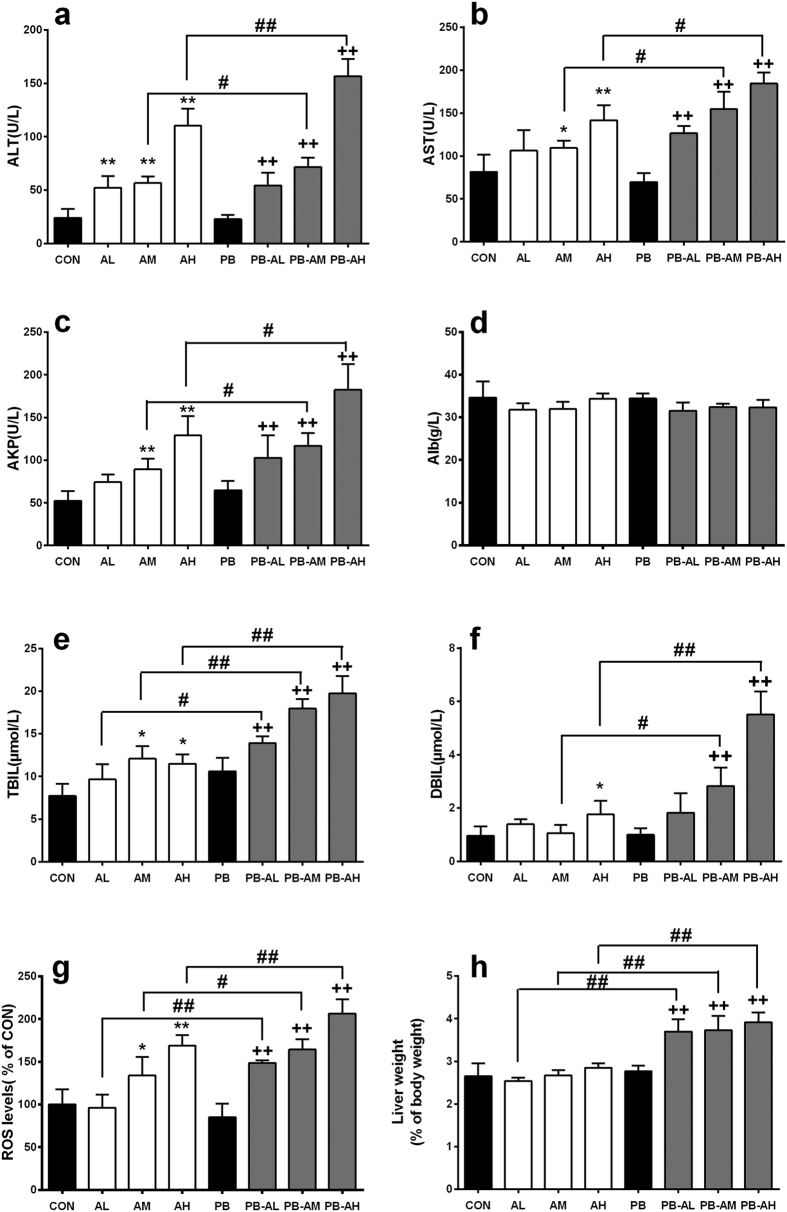
The serum levels of ALT (**a**), AST (**b**), AKP (**c**), Alb (**d**), TBIL (**e**), DBIL (**f**), ROS (**g**) and the liver weight (**h**) in normal rats (CON), normal rats treated with 10 mg/kg atorvastatin (AL), 20 mg/kg atorvastatin (AM) and 40 mg/kg atorvastatin (AH); normal rats treated with only phenobarbital (PB), rats co-administrated with phenobarbital and 10 mg/kg atorvastatin (PB-AL), 20 mg/kg atorvastatin (PB-AM) and 40 mg/kg atorvastatin (PB-AH). Data are represented as means ± SD (n = 5) *p < 0.05, **p < 0.01 vs. CON, ^+^p < 0.05, ^++^p < 0.01 vs. PB, ^#^p < 0.05, ^##^p < 0.01 vs. AL, AM or AH.

Rats pre-treated with phenobarbital(PB rats) were constructed to further verify the coupling roles of Cyp3a and SLCO1B1 in atorvastatin-induced hepatotoxicity (Fig. 6). The *in vivo* results demonstrated that atorvastatin exhibited mild hepatotoxicity in normal rats, while co-administrated with phenobarbital markedly potentiated the hepatotoxicity, significant increases in serum hepatotoxic biomarkers, production of ROS and liver weight were observed in PB-AL, PB-AM and PB-AH rats.

The *in vitro* study further demonstrated that atorvastatin induced severe cytotoxicity in primary hepatocytes of PB rats. The cell viability showed a concentration-denpendent decrease, the change was supported by the activity of LDH release (Fig. 3a,b). ROS level showed the same tendency as the cell viability (Fig. 3c). Co-incubated with antioxidants, SLCO1B1 inhibitors and Cyp3a inhibitor raised the cell viability and suppressed the production of ROS (Fig. 3g,h).

Activities and expressions of hepatic Cyp3a and SLCO1B1 in PB rats were also measured (Fig. 4). Treatment with phenobarbital significantly increased the activities and expressions of hepatic Cyp3a and SLCO1B1, which led to the enhanced uptake and metabolism of atorvastatin. These findings confirmed the contributions of the increased hepatic Cyp3a and SLCO1B1 activites to the enhanced atorvastatin-induced hepatotoxicity.

**Figure 7:**
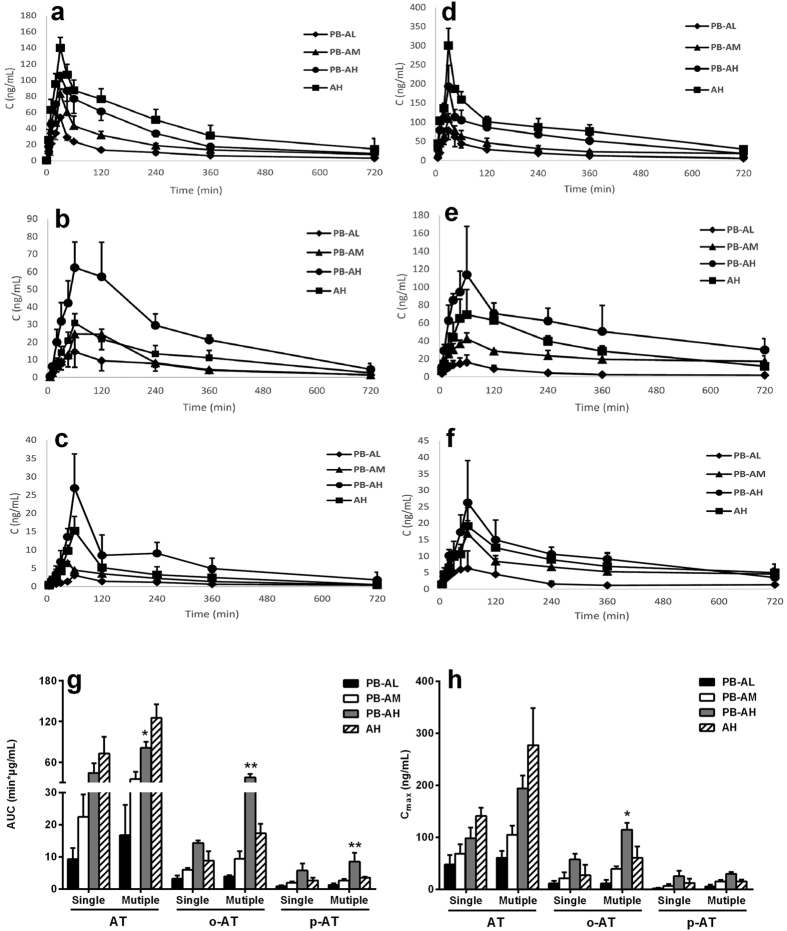
Plasma concentrations of atorvastatin (AT), ortho-hydroxyatorvastatin (o-AT) and para-hydroxyatorvastatin (p-AT) after single (**a–c**) or mutiple (**d–f**) oral dose (10 mg/kg ♦, 20 mg/kg ▲, 40 mg/kg ●) to PB rats and oral dose (40 mg/kg ■) to normal rats, respectively. The AUC (**g**) and C_max_ (**h**) levels of atorvastatin, ortho-hydroxyatorvastatin and para-hydroxyatorvastatin in normal and PB rats. Data are represented as means ± SD (n = 5). *p < 0.05, **p < 0.01 vs. AH.

To further investiagte effects of atorvastatin exposure on atorvastatin-induced hepatotoxicity, pharmackinetics of atorvastatin and its two metabolites following single and multiple doses of atorvastatin were measured (Fig. 7). It was was in line with atorvastatin-induced dose-denpendent hepatotoxicity that, the plasma exposure of atorvastatin and its metabolites increased along with the dose in PB rats. It was also noticed that the plasma exposure of atorvastatin in PB rats was lower than those in normal rats, accompanied by increases in exposure of its two metabolites. The estimated areas under the curve (AUC) of atorvastatin in PB rats following 11^th^ dose of atorvastatin (40 mg/kg) was significantly lower than that of normal rats (80.92 ± 9.09 μg*min/mL in PB rats versus 125.32 ± 20.00 μg*min/mL in normal rats, p = 0.021). C_max_ (194.09 ± 24.85 ng/mL in PB rats versus 277.01 ± 71.22 ng/mL in rats) of atorvastatin in PB rats showed a trend to increase, although no significance was observed. On contrast, C_max_ (114.35 ± 13.27 ng/mL p = 0.019) and AUC (37.64 ± 4.94 μg*min/mL, p = 0.0011) of ortho-atorvastatin following 11^th^ dose of atorvastatin (40 mg/kg) to PB rats were significantly higher than those of normal rats (C_max_ 60.25 ± 22.32 ng/mL and AUC 17.30 ± 2.97 μg*min/mL). Similar phenomena were observed in first dose study, but no significance was obtained. All these results demonstrated that both the decrease in plasma exposure of atorvastatin and the increases in plasma exposure of its metabolites may be partly attributed to the induction of hepatic Cyp3a and SLCO1B1.

## Discussion

The main finding of this study was that atorvastatin exhibited severe hepatotoxicity in diabetic rats. The *in vivo* toxicological experiment indicated that 40 mg/kg atorvastatin treatment was lethal to diabetic rats, whose mean survival time was 6.2 days. DM-AL and DM-AM rats also showed severe hepatic injury, as evidenced by significant alterations in serum hepatotoxic biomarkers (Fig. 1). The atorvastatin-induced hepatotoxicity was further confirmed by histological examination (Fig. 2).

*In vivo* findings were confirmed using primary rat hepatocytes, a reliable model for the toxicity study^18^. The results showed that atorvastatin-induced hepatotoxicity was dependent on physiological status. Atorvastatin induced hepatotoxicity in a concentration-dependent manner in primary hepatocytes of DM rats that is accompanied by a significant increase in ROS level. However, only high concentration of atorvastatin (30 μM) showed obvious hepatotoxicity in primary hepatocytes of CON rats.

Hepatic uptake of atorvastatin in rats is mediated by SLCO1B1, then it undergoes extensive metabolism by Cyp3a^19^. Several studies demonstrated that the expressions and activities of hepatic Cyp3a^16^,^20^ and SLCO1B1^16^,^17^ significantly upregulated in diabetic rats. Therefore, our study further investigated the coordinate activity of Cyp3a and SLCO1B1 in the aggravated hepatotoxicity of atorvastatin under diabetic status.

The uptake and metabolism experiment in primary rat hepatocytes demonstrated that the activity of Cyp3a and SLCO1B1 enhanced in DM rats, and the uptake and metabolic intrinsic clearance of atorvastatin increased by 2 folds and 3 folds, respectively (Fig. 4g). The Western blot analysis further illustrated that the protein expressions of hepatic Cyp3a and SLCO1B1 also increased in DM rats. In addition, co-incubation with SLCO1B1 inhibitor gemfibrozil and Cyp3a inhibitor ketoconazole significantly alleviated atorvastatin-induced hepatotoxicity in primary hepatocytes of DM rats (Fig. 3e). A high degree of correlation between hepatotoxicity and the activity of Cyp3a and SLCO1B1 were found and provided support for the roles of Cyp3a and SLCO1B1 in atorvastatin hepatotoxicity (Fig. 4h,i).

It was contrast to our expectation that two atorvastatin metabolites exhibited no hepatotoxicity, indicating that the hepatotoxic effect occurred during the metabolism. Previous reports have demonstrated that ROS production was associated with CYP-mediated metabolism^21^,^22^. A series of CYPs are susceptible to uncoupling of their catalytic cycle thus generating ROS and oxidative stress^13^. In this study, a significant increase in ROS level was observed in primary hepatocytes of DM rats after exposing to atorvastatin for 96 h (Fig. 3c). Antioxidants GSH and N-Acetylcysteine, Cyp3a inhibitor ketoconazole and SLCO1B1 inhibitor gemfibrozil suppressed both the hepatotoxicity and ROS formation (Fig. 3e,f). *In vivo* study also suggested that atorvastatin-induced hepatotoxicity was associated with oxidative stress, as evidenced by decreases in levels of SOD, CAT and GSH-pX as well as increases in levels of MDA and GSSG/T-GSH (Fig. 1g–l). ROS was also considered to be involved in the mechanisms of cellular toxicity and apoptosis^23^. All these results demonstrated that the increased production of ROS after Cyp3a and SLCO1B1 induction, at least, partly be responsible for the aggravated atorvastatin-induced hepatotoxicity under diabetic condition. Simliar mechanism was also found in other drug-induced hepatic injury, amiodarone was reported to induce apoptosis/necrosis in HepG2 cells via triggering the CYP3A4-mediated production of ROS^24^.

The mechanisms underlying the induction of hepatic Cyp3a and SLCO1B1 under diabetic condition has not been established. The increased levels of fatty acids were often associated with diabetes. Our previous study showed that serum from diabetic rats induced expression of CYP3A4 in HepG2 cells. Further study demonstrated that oleic acid and palmitic acid induced expressions of CYP3A4 and PXR in HepG2 cells^25^. Several studies demonstrated that PXR activation led to the upregulation of CYP3A4 and SLCO1B1^26^,^27^,^28^. All these results indicated that the increased levels of fatty acids upregulated the expressions of CYP3A4 and SLCO1B1 via PXR activation under diabetic status, which needed further investigation.

In human, hepatic uptake of atorvastatin and its subsequent metabolism were mainly mediated by SLCO1B1 and CYP3A4, respectively. Here, the *in vitro* results from primary rat hepatocytes were extrapolated to human using HepG2 cells. It was in line with our expectation that 72-h pre-incubation with CYP3A4/SLCO1B1 inducers rifampicin and phenobarbital significantly enhanced atorvastatin-induced hepatotoxicity, accompanied by increases in its metabolism and uptake as well as ROS generation. But, co-incubation with SLCO1B1 inhibitors (rifampicin and gemfibrozil) and CYP3A4 inhibitor (ketoconazole) showed the opposite effects (Fig. 5a,b). Further study demonstrated that CYP3A4 or SLCO1B1 knockdown attenuated atorvastatin hepatotoxicity and the increased ROS level in HepG2 cells, which was in line with the inhibition study (Fig. 5h,k). All these findings demonstrated the contributions of CYP3A4 and SLCO1B1 to atorvastatin-induced hepatotoxicity in HepG2 cells, which was consistent with the findings in primary rat hepatocytes.

Intraperitoneal administration of phenobarbital could induce the expressions and activities of hepatic Cyp3a and SLCO1B1 in rat^21^,^29^. Therefore, this model was used to further confirm above results. It was consistent with the findings in DM rats that the hepatotoxicity, metabolism and uptake of atorvastatin and ROS formation in primary hepatocytes of PB rats were significantly increased (Figs 3 and 4). GSH and N-Acetylcysteine, gemfibrozil and ketoconazole all reversed the cytotoxicity and ROS level (Fig. 3g,h). The *in vivo* study showed that atorvastatin induced mild hepatic injury in normal rats, coadministration with phenobarbital significantly potentiated atorvastatin-induced hepatotoxicity. The alterations in serum hepatotoxic biomarkers by coadiministrated with phenobarbital were similar to those in DM rats, although the extent was less (Fig. 6). Further study showed that althouth plasma exposure of atorvastatin in PB rats were lower than those in normal rats (Fig. 7), atorvastatin showed stronger hepatotoxicity in PB rats than that in normal rats. All these results indicated that phenobarbital aggravated atorvastatin hepatotoxicity via inducing expression of Cyp3a and SLCO1B1 and increasing atorvastatin metabolism, which further demonstrated the roles of the upregulated hepatic SLCO1B1 and Cyp3a in the enhanced atorvastatin-induced hepatotoxicity.

Skeletal muscle toxicity was another severe side-effect of atorvastatin. SLCO1B1 and SLCO2B1 were reported to be expressed in skeletal muscles^30^. A report demonstrated involvement of muscular SLCO1B1 in fluvastatin-induced muscle toxicity^30^. However, whether SLCO1B1 was also induced by diabetes was not identified. In addition, clinical trials showed that statins-induced myopathy was also related to the system exposure of statins^31^. Our previous reports showed that diabetes decreased system exposure of atorvastatin in rats^32^. Here we also reported that phenobarbital treatment decreased plasma exposure of atorvastatin in rats via inducing expressions of hepatic Cyp3a and SLCO1B1, but it still aggravated atorvastatin hepatotoxicity. Therefore, whether the decrased plasma exposure of atorvastatin affected atorvastatin-induced myopathy under diabetic condition needed to be further investigated.

In conclusion, the present study clearly demonstrated that the increased expressions and activities of hepatic Cyp3a and SLCO1B1 under diabetic status markedly enhanced the hepatotoxicity of atorvastatin via increasing formation of ROS during metabolism. Although the data cannot be directly extrapolated to human, attentions should still be paid to the higher susceptibility of atorvastatin-induced hepatotoxicity in diabetic patients.

## Materials and Methods

### Reagents

Repaglinide and midazolam from the National Institute for the Control of Pharmaceutical and Biological Products (Beijing, China). Atorvastatin calcium and gemfibrozil from Dalian Meilun Biology Technology Co, Ltd (Dalian, China). Ketoconazole and rifampicin from J&K Chemical (Shanghai, China). 1-hydroxymidazolam from Cayman Chemical (Ann Arbor, MI, USA). Phenobarbital from New Asia Pharmaceutical Company (Shanghai, China). Streptozotocin (STZ) and 2′,7′-dichlorofluorescin diacetate (DCFH-DA) from Sigma Chemical Co (St Louis, MO, USA). Para-hydroxy atorvastatin calcium and ortho-hydroxy atorvastatin calcium from TLC PharmaChem Inc. (Canada). Glutathione (GSH) and N-acetylcystiene from Sunshine Biotechnology Co. Ltd (Nanjing, China). Mouse monoclonal Cyp3a, CYP3A4 and SLCO1B1 antibodies from Santa Cruz Technology (Santa Cruz, CA, USA). GAPDH antibody from Bioworld (Louis Park, MN, USA). Silencer^®^ Pre-Designed siRNA (CYP3A4), Silencer^®^ Pre-Designed siRNA (SLCO1B1), Silencer^®^ Negative Control siRNA, Lipofectamine1 RNAiMAX Transfection Reagent and Opti-MEM^®^ I Reduced Serum Medium were purchased from Molecular Probes (Invitrogen, Burlington, Ontario, Canada). All the other reagents were commercially available.

### Animals

Male Sprague-Dawley rats (weighing 100–120 g), purchased from Sino-British Sipper & BK Lab Animal Ltd. (Shanghai, China), housed in an environment of controlled temperature (22 ± 2 °C) and relative humidity (50% ± 5%) with 12 h light/darkness cycle. Water and food were allowed *ad libitum*. Use of animals and experimental procedures were conducted in accordance with the Guide for the Care and Use of Laboratory Animals published by the US National Institutes of Health (NIH publication 86-23, revised 1986), and their use was approved by the Animal Ethics Committee of China Pharmaceutical University (No. CPU-PCPK-13211010345).

### Induction of diabetic rats and drug treatment

Diabetic rats were induced by the combination of high-fat diet feeding and low-dose STZ injection according to method described previously with some modifications^33^,^34^. Briefly, experimental rats were assigned randomly to three groups: control (CON) group (n = 5), high-fat diet (HFD) group (n = 5) and diabetic (DM) group (n = 20). The CON rats were fed on normal chow while both HFD rats and DM rats were fed on high-fat diet (TROPHIC Animal Feed High-tech Co. Ltd, Nantong, China). After six-week of dietary manipulation, DM rats intraperitoneally (i.p.) received a single dose of STZ (35 mg/kg). On day 7 after vehicle or STZ injection, Only rats with FBG level higher than 11.1 mM were considered as successful diabetic models and were selected for following studies^33^.

The diabetic rats were randomly divided into four groups (n = 5 for each group). The first group served as control group (DM). The other three groups served as atorvastatin-treated groups, which orally received low-dose (10 mg/kg, DM-AL), middle-dose (20 mg/kg, DM-AM) and high-dose (40 mg/kg, DM-AH) of atorvastatin once daily for 10 days. The dose of atorvastatin in rats was referenced from previous reports^35^,^36^. Meanwhile, CON rats, HFD rats and DM rats received vehicle (0.25% CMC-Na).

Following last dose, rats, fasted overnight, were anesthetized with diethyl ether and blood samples were collected for measurement of blood chemistry. Then, the animals were sacrificed; the liver samples were harvested for standard histology, hepatic biochemical measurement and Western blot.

### Liver histological examination

The livers were sliced into 0.3–0.5 cm blocks and immersed in 10% formalin solution. After dehydration, the liver blocks were embedded in paraffin. Liver sections (4 μm) were cut, stained with haematoxylin-eosin. Each stained section was semi-quantitatively evaluated under light microscope (Inverted Microscope ECLIPSE Ti-S, Nikon, Japan) by an investigator blind to the experimental group. The severity of hepatic injury was assessed according to the extent of: (1) vacuolization, (2) inflammatory cell infiltration, (3) pyknotic hepatocyte nuclei, (4) sinusoidal dilatation, which was scored as 0, no response; 1, mild response; 2, moderate response and 3, severe response. The maximum score was 12, indicating the most severe hepatic injury^37^,^38^.

### Determination of physiological and biochemical parameters

Plasma glucose level, total cholesterol (TG), triglyceride (TC), serum hepatotoxic biomarkers including alkaline phosphatase (AKP), alanine aminotransferase (ALT), aspartate aminotransferase (AST), albumin (Alb), direct bilirubin (DBIL) and total bilirubin (TBIL); and hepatic oxidative stress-related biomarkers including succinate dehydrogenase (SDH), superoxide dismutase (SOD), catalase (CAT), glutathione peroxidase (GSH-pX), GSSG (oxidized glutathione), total glutathione (T-GSH) and malondialdehyde (MDA), were measured by commercial reagent kits (Jiancheng Bioengineering Institute, Nanjing, China) according to the instructions.

### Cell culture

The HepG2 cells were obtained from Shanghai Cellular Research Institute (Shanghai, China). The cells were cultured in DMEM containing 10% FBS (fetal bovine serum), non-essential amino acids, antibiotics (100 IU/ml penicillin and 100 μg/ml streptomycin), 2 mM L-glutamine, and 3.7 g/L NaHCO_3_ in a humidified incubator of 5% CO_2_ and 95% air atmosphere at 37 °C.

Another subset of experimental rats (CON, HFD and DM rats) were used for the primary rat hepatocytes isolation. CON rats pre-treated with phenobarbital (PB) were simultaneously obtained via intraperitoneally injected phenobarbital (80 mg/kg) for 5 days. Primary hepatocytes were isolated from experimental rats in a modified two-step collagenase perfusion method as previously reported^17^,^39^. An aliquot of the hepatocyte suspension (at a density of 2.5 × 10^5^/ml) was seeded in collagen-precoated plates. Following 4-h incubation, the medium was replaced with 500 μl fresh cultured medium. Twenty-four hours after plating, the medium was removed and the hepatocytes were overlaid with BD matrigel (0.25 mg/ml) in feeding medium (DMEM containing 0.1 mM dexamethasone and 1% ITS) and cultured for another 24 h at 37 °C in a humidified atmosphere containing 5% CO_2_ for following experiments.

### Hepatotoxicity of atorvastatin in primary rat hepatocytes

Our preliminary results showed that the hepatotoxicity was time-dependent and the strongest hepatotoxicity occurred after 96 h incubation. Thus, the incubation time was set to be 96 h. The primary rat hepatocytes were exposed to atorvastatin (2, 10 and 30 μM) or hydroxy atorvastatin (0.5, 1 and 10 μM) for 96 h. Cell viability was determined using both the 3-(4,5-dimethyl-2-thiazolyl)-2,5-diphenyl-2-H-tetrazolium bromide (MTT) assay^40^ and lactate dehydrogenase (LDH) release measurement. ROS generation was simultaneously measured using DCFH-DA as a fluorescent probe. Effects of GSH (0.5 and 2 mM) and N-Acetylcysteine (0.5 and 2 mM), ketoconazole (2 μM) and gemfibrozil (10 and 50 μM) on the atorvastatin-induced (10 μM) cytotoxicity and formation of ROS in primary hepatocytes of DM rats and PB rats were further investigated.

### Uptake and metabolism experiments in primary rat hepatocytes

The primary hepatocytes were washed twice with warm Hank’s balanced salt solution (HBSS) and pre-incubated with warm HBSS for 5 min. The reaction was initiated by adding 500 μL HBSS containing various concentrations of atorvastatin (0.1, 0.5, 2, 10 and 40 μM). For uptake experiment, the reaction was stopped after 2 min incubation by washing with ice-cold HBSS for three times; for metabolism experiment, the reaction was terminated after 30 min incubation by washing with ice-cold HBSS for three times.

The intracellular concentrations of atorvastatin, ortho- and para-hydroxy atorvastatin were measured using the LC-MS method^32^. Repaglinide (20 μM) and midazolam (10 μM) were used as probe substrates to identify the activities of SLCO1B1 and Cyp3a, respectively^17^.

### Hepatotoxicity of atorvastatin in HepG2 cells

The cells were pre-incubated with CYP3A4/SLCO1B1 inducers phenobarbital (0.5 and 2 mM) or rifampicin (10 and 25 μM) for 72 h. Following washing twice with fresh medium, HepG2 cells were exposed to atorvastatin (10 μM) for another 24 h, cell viability and ROS generation were measured using MTT assay and fluorescent probe, respectively. Effects of CYP3A4 inhibitor and SLCO1B1 inhibitors on ROS generation and hepatotoxicity by atorvastatin in HepG2 cells were also documented. Briefly, HepG2 cells were co-incubated with atorvastatin (30 μM) and ketoconazole (2 and 10 μM), gemfibrozil (10 and 50 μM) or rifampicin (10 and 25 μM) for 24 h, the cell viability and ROS generation were measured.

### Effect of siRNA-mediated knockdown of CYP3A4 and SLCO1B1 on atorvastatin hepatotoxicity in HepG2 cells

HepG2 cells were transfected with siRNA using Lipofectamine RNAiMAX, human CYP3A4 siRNA and SLCO1B1 siRNA according to the manufacturer’s instructions. After culturing for 48 h, cells were incubated with atorvastatin (30 μM) for another 24 h. LDH release and ROS generation were measured. Efficiencies of knockdown on CYP3A4 and SLCO1B1 were confirmed by Western blots.

### Atorvastatin uptake and metabolism in HepG2 cells

For effects of CYP3A4/SLCO1B1 inducers on atorvastatin uptake and metabolism in HepG2 cells, following 72-h exposure to phenobarbital (0.5 and 2 mM) or rifampicin (10 and 25 μM), 2-min uptake and 30-min metabolism (indexed as the formation of ortho-hydroxy atorvastatin) of atorvastatin (30 μM) in HepG2 cells were assessed^32^. For effects of CYP3A4 inhibitor and SLCO1B1 inhibitors on atorvastatin uptake and metabolism in HepG2 cells, following 5-min pre-incubation with ketoconazole (2 and 10 μM), rifampicin (10 and 25 μM) or gemfibrozil (10 and 50 μM), 2-min uptake and 30-min metabolism of atorvastatin in HepG2 cells were measured by co-incubation atorvastatin (30 μM) with the tested inhibitors as described above. Repaglinide and midazolam were used to index the activities of SLCO1B1 and CYP3A4, respectively^17^.

### Effect of phenobarbital on atorvastatin-induced hepatotoxicity

Atorvastatin-induced hepatotoxicity was further investigated in normal rats and rats pre-treated with phenobarbital. For normal rats, they were randomly divided into vehicle (CON) rats, low-dose treatment (AL) rats, middle-dose treatment (AM) rats and high-dose treatment (AH) rats (n = 5 for each group). CON rats orally received daily dose of vehicle. AL rats, AM rats and AH rats orally received 10 mg/kg, 20 mg/kg and 40 mg/kg of atorvastatin for 10 days. For phenobarbital co-treated rats, following 3-day pre-treatment with phenobarbital (i.p. 80 mg/kg), rats co-administrated phenobarbital (i.p.80 mg/kg) with oral daily dose of vehicle (PB), 10 mg/kg (PB-AL), 20 mg/kg (PB-AM), and 40 mg/kg (PB-AH) atorvastatin for another 10 days, respectively. The serum biochemical parameters, liver weight and ROS levels were measured as described above.

### Pharmacokinetics of atorvastatin following oral administration to normal and phenobarbital pretreated rats

Pharmacokinetics of atorvastatin following single and multiple oral administrations were documented. Twenty male rats were randomly divided into normal rats and phenobarbital co-treated (PB) rats with atorvastatin. For PB rats, following 3-day pre-treatment with phenobarbital (i.p. 80 mg/kg), the PB rats co-administrated phenobarbital (i.p. 80 mg/kg) with 10 mg/kg (PB-AL), 20 mg/kg (PB-AM), and 40 mg/kg (PB-AH) of atorvastatin for another 11 days (n = 5 for each group), respectively. Normal rats (n = 5) only received 40 mg/kg of atorvastatin for 11 days. Blood samples were collected under light ether anesthesia via the oculi chorioideae vein at 5, 10, 20, 30, 45, 60, 120, 240, 360, and 720 min after first dose and last dose. After 3 or 4 samplings, the appropriate amount of normal saline was administered to the rats via the tail vein to compensate for blood loss. The blood collected was mixed properly with the anticoagulant and centrifuged at 4000 rpm for 10 min. The plasma was separated and stored at −80 °C until drug analysis. Plasma concentrations of atorvastatin and its metabolites were measured using the LC-MS method as described previously^32^.

### Western blot analysis

Total rat liver extract was prepared in RIPA lysis buffer containing 1 mM PMSF to measure protein levels of Cyp3a and SLCO1B1. Protein concentrations were measured by the BCA protein assay kit. 50 μg proteins were separated on a SDS-polyacrylamide gel electrophoresis and transferred onto polyvinylidene fluoride membranes (Millipore Corporation, Billerica, MA, USA). These blots were blocked for 2 h with 5% non-fat dry milk in 10 mM Tris-buffered saline containing 0.1% Tween 20 and then washed. The membranes were then incubated overnight at 4 °C with given primary antibodies, following by HRP-conjugated secondary anti-mouse antibody incubation for 2 h. Protein quantification was performed by Tanon 5200 Multi Chemiluminescent System (Tanon technology Co., Ltd., Shanghai, China). Intensity values were normalized to the quantity of GAPDH for total protein.

### Statistical analysis

The pharmacokinetic parameters were calculated using noncompartmental analysis on Phenix WinNonlin 6.1 (Pharsight, St. Louis, MO). All values were expressed as mean ± S.D. Results were analyzed using one-way analysis of variance (ANOVA) followed by Tukey’s post hoc test. Differences among groups were determined by nonparametric test when necessary. Statistical significance was assumed at P < 0.05.

## Additional Information

**How to cite this article**: Shu, N. *et al*. The enhanced atorvastatin hepatotoxicity in diabetic rats was partly attributed to the upregulated hepatic Cyp3a and SLCO1B1. *Sci. Rep.* **6**, 33072; doi: 10.1038/srep33072 (2016).

## Figures and Tables

**Table 1:** Physiological and biochemical characteristics in experiment rats.

Parameter	CON	HFD	DM	DM-AL	DM-AM
Body weight (g)	239.3 ± 12.2	305.3 ± 10.2**	290.2 ± 20.6**	269.3 ± 5.3**	250.8 ± 22.4
Liver weight (% of body weight)	2.85 ± 0.16	4.71 ± 0.22**	5.46 ± 0.27**	6.94 ± 0.95**^##^	10.60 ± 2.25**^##^
Spleen weight (% of body weight)	0.198 ± 0.027	0. 215 ± 0.010	0.218 ± 0.024	0.264 ± 0.027**^#^	0.392 ± 0.033**^##^
FBG (mM)	7.53 ± 0.34	7.76 ± 0.65	16.92 ± 1.35**	18.38 ± 1.75**	18.34 ± 1.60**
FINS (mIU/L)	29.20 ± 3.06	30.12 ± 2.41	34.42 ± 3.99	32.65 ± 2.15	32.51 ± 4.45
HOMA-IR	9.56 ± 1.05	10.32 ± 1.25	25.51 ± 1.79**	27.56 ± 2.01**	26.92 ± 2.52**
TC (mM)	1.88 ± 0.14	2.82 ± 0.72*	7.98 ± 1.64**	4.02 ± 0.71**^##^	3.42 ± 0.52**^##^
TG (mM)	1.23 ± 0.14	2.56 ± 0.30**	7.00 ± 1.76**	5.40 ± 0.50**	3.75 ± 0.70**^##^

Data are represented means ± SD (n = 5) *p < 0.05, **p < 0.01 vs. CON, ^#^p < 0.05, ^##^p < 0.01 vs. DM rats. Symbols: CON, normal rats; HFD, high-fat diet fed rats; DM, diabetic rats; DM-AL, diabetic rats treated with 10 mg/kg atorvastatin and DM-AM, diabetic rats treated with 20 mg/kg atorvastatin.

## References

[b1] Colhoun H. M. . Primary prevention of cardiovascular disease with atorvastatin in type 2 diabetes in the Collaborative Atorvastatin Diabetes Study (CARDS): multicentre randomised placebo-controlled trial. Lancet 364, 685–696 (2004). 15325833 10.1016/S0140-6736(04)16895-5

[b2] Wang C. Y., Liu P. Y. & Liao J. K. Pleiotropic effects of statin therapy: molecular mechanisms and clinical results. Trends Mol Med. 14, 37–44 (2008). 18068482 10.1016/j.molmed.2007.11.004 PMC2621332

[b3] Bays H. Statin safety: an overview and assessment of the data—2005. Am J Cardiol. 97, S6–S26 (2006). 10.1016/j.amjcard.2005.12.006 16581330

[b4] Björnsson E., Jacobsen E. I. & Kalaitzakis E. Hepatotoxicity associated with statins: reports of idiosyncratic liver injury post-marketing. J. Hepatol. 56, 374–380 (2012). 21889469 10.1016/j.jhep.2011.07.023

[b5] Pasternak R. C. . ACC/AHA/NHLBI clinical advisory on the use and safety of statins12. J. Am. Coll. Cardiol. 40, 567–572 (2002). 12142128 10.1016/s0735-1097(02)02030-2

[b6] Kromer A. & Moosmann B. Statin-induced liver injury involves cross-talk between cholesterol and selenoprotein biosynthetic pathways. Mol Pharmacol. 75, 1421–1429 (2009). 19332511 10.1124/mol.108.053678

[b7] Tavintharan S. . Reduced mitochondrial coenzyme Q10 levels in HepG2 cells treated with high-dose simvastatin: a possible role in statin-induced hepatotoxicity? Toxicol Appl Pharmacol. 223, 173–179 (2007). 17610923 10.1016/j.taap.2007.05.013

[b8] Mullen P. J. . Susceptibility to simvastatin-induced toxicity is partly determined by mitochondrial respiration and phosphorylation state of Akt. BBA Mol Cell Res. 1813, 2079–2087 (2011). 10.1016/j.bbamcr.2011.07.019 21839782

[b9] Black A. E., Hayes R. N., Roth B. D., Woo P. & Woolf T. F. Metabolism and excretion of atorvastatin in rats and dogs. Drug Metab Dispos. 27, 916–923 (1999). 10421619

[b10] Kantola T., Kivistö K. T. & Neuvonen P. J. Effect of itraconazole on the pharmacokinetics of atorvastatin. Clin Pharmacol Ther. 64, 58–65 (1998). 9695720 10.1016/S0009-9236(98)90023-6

[b11] Shitara Y. . Clinical significance of organic anion transporting polypeptides (OATPs) in drug disposition: their roles in hepatic clearance and intestinal absorption. Biopharm Drug Dispos 34, 45–78 (2013). 23115084 10.1002/bdd.1823

[b12] White R. E. & Coon M. J. Oxygen activation by cytochrome P-4501. Annu. Rev. Biochem. 49, 315–356 (1980). 6996566 10.1146/annurev.bi.49.070180.001531

[b13] Nesnow S. . Propiconazole increases reactive oxygen species levels in mouse hepatic cells in culture and in mouse liver by a cytochrome P450 enzyme mediated process. Chem. Biol. Interact. 194, 79–89 (2011). 21864511 10.1016/j.cbi.2011.08.002

[b14] Guengerich F. P. . Oxidation of dihydropyridine calcium channel blockers and analogs by human liver cytochrome P-450 IIIA4. J. Med. Chem. 34, 1838–1844 (1991). 2061924 10.1021/jm00110a012

[b15] Nobuo S. Cytochrome P450 changes in rats with streptozocin-induced diabetes. Int J Biochem Cell B. 26, 1261–1268 (1994). 10.1016/0020-711x(94)90095-7 7880321

[b16] Xu D. . Decreased exposure of simvastatin and simvastatin acid in a rat model of type 2 diabetes. Acta Pharmacol. Sin. 9, 1215–1225 (2014). 25152023 10.1038/aps.2014.39 PMC4155525

[b17] Li F. . Co-administration of paroxetine and pravastatin causes deregulation of glucose homeostasis in diabetic rats via enhanced paroxetine exposure. Acta Pharmacol. Sin. 35, 792–805 (2014). 24902787 10.1038/aps.2014.24 PMC4086381

[b18] Tuschl G. & Mueller S. O. Effects of cell culture conditions on primary rat hepatocytes—cell morphology and differential gene expression. Toxicology 218, 205–215 (2006). 16337326 10.1016/j.tox.2005.10.017

[b19] Chang J. H. . Differential effects of Rifampin and Ketoconazole on the blood and liver concentration of atorvastatin in wild-type and Cyp3a and Oatp1a/b knockout mice. Drug Metab Dispos. 42, 1067–1073 (2014). 24671957 10.1124/dmd.114.057968

[b20] Shimojo N. . Changes in amounts of cytochrome P450 isozymes and levels of catalytic activities in hepatic and renal microsomes of rats with streptozocin-induced diabetes. Biochem. Pharmacol. 46, 621–627 (1993). 8363636 10.1016/0006-2952(93)90547-a

[b21] Minamiyama Y. . CYP3A induction aggravates endotoxemic liver injury via reactive oxygen species in male rats. Free Radical Bio Med. 37, 703–712 (2004). 15288127 10.1016/j.freeradbiomed.2004.05.022

[b22] Lu Y., Meng Q., Zhang G. & Bei X. Clozapine-induced hepatotoxicity in rat hepatocytes by gel entrapment and monolayer culture. Toxicol In vitro 22, 1754–1760 (2008). 18761400 10.1016/j.tiv.2008.08.002

[b23] Sena L. A. & Chandel N. S. Physiological roles of mitochondrial reactive oxygen species. Mol cell. 48, 158–167 (2012). 23102266 10.1016/j.molcel.2012.09.025 PMC3484374

[b24] Zahno A. . The role of CYP3A4 in amiodarone-associated toxicity on HepG2 cells. Biochem. Pharmacol. 81, 432–441 (2011). 21070748 10.1016/j.bcp.2010.11.002

[b25] Hu N. . Increased levels of fatty acids contributed to induction of hepatic CYP3A4 activity induced by diabetes-*in vitro* evidence from HepG2 cell and Fa2N-4 cell lines. J. Pharmacol. Sci. 124, 433–444 (2014). 24739263 10.1254/jphs.13212fp

[b26] Sakurai M., Saito F., Ohata Y., Yabe Y. & Nishi T. Organic anion transporting polypeptides (OATPs): regulation of expression and function. Curr Drug Metab 12, 139–153 (2011). 21395542 10.2174/138920011795016863

[b27] Doricakova A. & Vrzal R. A food contaminant ochratoxin A suppresses pregnane X receptor (PXR)-mediated CYP3A4 induction in primary cultures of human hepatocytes. Toxicology 337, 72–78 (2015). 26341324 10.1016/j.tox.2015.08.012

[b28] Tamai I. Oral drug delivery utilizing intestinal OATP transporters. Adv drug deliver rev. 64, 508–514 (2012). 10.1016/j.addr.2011.07.007 21824501

[b29] Hagenbuch N. . Effect of phenobarbital on the expression of bile salt and organic anion transporters of rat liver. J Hepatol. 34, 881–887 (2001). 11451172 10.1016/s0168-8278(01)00097-6

[b30] Sakamoto K., Mikami H. & Kimura J. Involvement of organic anion transporting polypeptides in the toxicity of hydrophilic pravastatin and lipophilic fluvastatin in rat skeletal myofibres. Br. J. Pharmacol. 154, 1482–1490 (2008). 18500364 10.1038/bjp.2008.192 PMC2492093

[b31] Holbrook A., Wright M., Sung M., Ribic C. & Baker S. Statin-associated rhabdomyolysis: is there a dose-response relationship? Can. J. Cardiol. 27, 146–151 (2011). 21459261 10.1016/j.cjca.2010.12.024

[b32] Shu N. . Decreased exposure of atorvastatin in diabetic rats partly due to induction of hepatic Cyp3a and Oatp2. Xenobiotica 46, 875–881 (2016). 26864241 10.3109/00498254.2016.1141437

[b33] Liu C. . Increased glucagon-like peptide-1 secretion may be involved in antidiabetic effects of ginsenosides. J. Endocrinol. 217, 185–196 (2013). 23444389 10.1530/JOE-12-0502

[b34] Ji J. . Effect of Stay-Green Wheat, a Novel Variety of Wheat in China, on Glucose and Lipid Metabolism in High-Fat Diet Induced Type 2 Diabetic Rats. Nutrients 7, 5143–5155 (2015). 26132991 10.3390/nu7075143 PMC4516991

[b35] Elewa H. F. . Early atorvastatin reduces hemorrhage after acute cerebral ischemia in diabetic rats. J. Pharmacol. Exp. Ther. 330, 532–540 (2009). 19478137 10.1124/jpet.108.146951 PMC2713088

[b36] Van Linthout S. . Anti-inflammatory effects of atorvastatin improve left ventricular function in experimental diabetic cardiomyopathy. Diabetologia 50, 1977–1986 (2007). 17589825 10.1007/s00125-007-0719-8

[b37] Akbulut S. . Cytoprotective effects of amifostine, ascorbic acid and N-acetylcysteine against methotrexate-induced hepatotoxicity in rats. World J Gastroentero. 20, 10158–10165 (2014). 10.3748/wjg.v20.i29.10158 PMC4123346 25110444

[b38] Dinis-Oliveira R. . Single high dose dexamethasone treatment decreases the pathological score and increases the survival rate of paraquat-intoxicated rats. Toxicology 227, 73–85 (2006). 16956706 10.1016/j.tox.2006.07.025

[b39] Kemp D. C. & Brouwer K. L. Viability assessment in sandwich-cultured rat hepatocytes after xenobiotic exposure. Toxicol In vitro 18, 869–877 (2004). 15465654 10.1016/j.tiv.2004.04.014

[b40] Mosmann T. Rapid colorimetric assay for cellular growth and survival: application to proliferation and cytotoxicity assays. J. Immunol. Methods. 65, 55–63 (1983). 6606682 10.1016/0022-1759(83)90303-4

